# Breaking bad news protocol for cancer disclosure: an Iranian version

**Published:** 2017-12-19

**Authors:** Parvaneh Abazari, Fariba Taleghani, Simin Hematti, Azadeh Malekian, Fariborz Mokarian, Sayyed Mohammad Reza Hakimian, Maryam Ehsani

**Affiliations:** 1 *Assistant Professor, Nursing and Midwifery Care Research Center, Faculty of Nursing and Midwifery, Isfahan University of Medical Sciences, Isfahan, Iran. *; 2 *Professor, Nursing and Midwifery Care Research Center, Faculty of Nursing and Midwifery, Isfahan University of Medical Sciences, Isfahan, Iran. *; 3 *Associate Professor, Department of Radiotherapy and Oncology, Faculty of Medicine, Isfahan University of Medical Sciences, Isfahan, Iran. *; 4 *Researcher, Psychosomatic Research Center, Isfahan University of Medical Sciences, Isfahan, Iran. *; 5 *Assistant Professor, Cancer Prevention Research Center, Isfahan University of Medical Sciences, Isfahan, Iran. *; 6 *Cancer Prevention Research Center, Isfahan University of Medical Sciences, Isfahan, Iran. *; 7 *Assistant Professor, Nursing Department, Islamic Azad University Complex, Tonekabon, Iran.*

**Keywords:** *Breaking bad news*, *Protocol*, *Cancer disclosure*, *Iranian version*

## Abstract

In Iran, as in many Asian and Middle Eastern countries, a significant proportion of cancer patients are never informed of their illness. One solution that has been proposed to tackle this challenge is to develop a localized protocol on cancer diagnosis disclosure based on the culture and values of community members, and train healthcare team members to deliver the bad news using this protocol. This article introduces a localized protocol for disclosure of bad news to cancer patients in Iran. This protocol consists of six steps, including assessment, planning, preparation, disclosure, support and conclusion.

In the drafting of this protocol an attempt was made to meticulously consider the cultural features of the Iranian society. Although breaking bad news will never be easy, having an appropriate plan of action based on individual’s attitudes, considerably helps health-care professionals, and provides more satisfaction in patients.

## Introduction

Today, in many Western countries, honest and full disclosure of medical information is a common and accepted procedure, and since standards of medical ethics lay great emphasis on patients’ rights and their independence, physicians deliver bad news to their patients honestly and directly (1, 2). In fact, disclosure of bad news is no more a matter of concern in Western countries, and the only important issue is the best way in which the healthcare team should deliver the news; In many Asian countries, however, the phenomenon of breaking bad news is faced with several challenges, as there is still no consensus on whether bad news should be delivered to the patients or not (3, 4). 

In Iran, studies show that around 40 percent of patients do not get informed of their disease (5). While the majority of Iranian cancer patients prefer to be aware of the nature of their disease, they do not get informed (6, 7) 

Regarding the importance and necessity of bad news disclosure, many researchers today are looking for patterns to help them with proper and scientific implementation of this task with the least emotional and psychological impact on patients and their families (8). Adopting a systematic strategy for breaking bad news is a demanding task. The lack of such a strategy or a roadmap to control patients’ reactions when disclosing the news may cause physicians not to reveal all aspects of the news or even to give false hope to patients. This would decrease patients’ confidence in physicians and make them unwilling to participate in clinical decisions (9). Despite the fact that such protocols and guidelines have been used in Western countries for a long time to train healthcare teams for bad news disclosure, in many Asian countries, including Iran, there are no such protocols in effect. Since bad news disclosure or truth telling is a purely cultural issue bearing on the values and beliefs of every single society (1, 4, 8, 10-15), various studies conducted in Iran commonly emphasize the necessity of developing a localized protocol for bad news disclosure and recognize the lack of such a protocol as an obstacle in effective implementation of the procedure (7, 8, 13, 16, 17).

Considering the importance of local and culture-based guidelines for disclosure of bad news in an effective way, this review article aims to introduce a protocol based on the Iranian culture to disclose cancer diagnosis to patients. 

## Method

The protocol introduced in this paper is part of the last author’s PhD dissertation, which was developed using a consecutive mixed-methods (qualitative-quantitative) study (18-20). 

*Truth Telling Protocol for Cancer Patients*

This protocol is developed for breaking the bad news of cancer diagnosis to cancer patients and their family members, and includes six steps: assessment, planning, preparation, disclosure, support, and conclusion. The minimum members comprising the bad news disclosure team are: an oncologist or surgeon, a nurse trained in care of cancer patients, and a clinical psychologist. 

*1)    Assessment*

This step is run by a nurse along with a psychologist, and the patients and one of their closest family members provide the required basic information on the individual characteristics of the patients. The information would include education, occupation, religious beliefs, history of psychological diseases and receiving psychiatric drugs, patients’ desire to get informed about their illness, and the willingness of family members to provide the patients with such information. The information is assessed by the nurse, except for the history of psychological diseases and how the patients have dealt with the previous challenges in their life. One of the main duties of the nurse at this stage is to clarify the following:

1. Does the patient wish to be informed of the diagnosis?

2. If the answer to the first question is “Yes”, which one of his/her family members does he/she prefer to be advised of the diagnosis as well? 

3. If the answer to the first question is “No” (i.e., the patient is not willing to receive this information), who does he/she prefer to have this information?

4. Does the closest family member of the patient wish to disclose the diagnosis to the the patient when the latter is willing to receive it?

*2)    Planning*

Based on the patients’ answers and the reactions of their closest family members to the questions raised in the previous step, three situations may occur for which the nurse has to plan in advance:

*a.* *Both the patient and their closest family member wish to get informed of the diagnosis:* In this situation there is no challenge in telling the truth to them and the nurse only needs to provide the required conditions for the “truth-telling” session, and without going through the "family preparation" step, will directly enter the "environment preparation" step.

*b. The patient wishes to learn about the diagnosis, but the family is reluctant to tell the truth:* This is a relatively difficult situation that is very common in the culture of Asian countries and the Middle East, including Iran. In this case, and before preparing the requirements for “truth telling”, the nurse has to arrange a meeting with the patient’s family to obtain their consent and convince them of the necessity of telling the truth to the patient, so that she can perform the other steps of the protocol. 

*c. The patient is not willing to know about his/her illness and prefers that other family members receive the information:* Patients should be assured that information on their diagnosis would be disclosed to them at their preferred time and to the extent that they choose. In this case, the nurse should make arrangements for the truth-telling session with the patient’s close family member(s), so that at first the "environment preparation" and then the other protocol steps are performed. It should be noted that in situation (c) after the "environment preparation" stage, all protocol steps should be performed focusing on the patient’s close family member(s), since the patient would not attend the truth-telling session in accordance with his/her request. 

*3)    Preparation*

The preparation step consists of three sub steps: family preparation, environment preparation, and patient preparation. 

*a. Family preparation*

If the data obtained in the “assessment” step are indicative of situation (b), it is necessary to arrange a "family preparation" session. This session should be held in the room designated for truth-telling, or if necessary, in the physician’s office in the ward, in the presence of a close family member and the nurse. The main purpose of this session is to investigate the reasons behind the family’s opposition by breaking the cancer news to the patient as well as persuading the former of the necessity of disclosing the diagnosis to the patient in an appropriate and proper way. It is essential for this room to be in a suitable condition, for instance there should be chairs, drinking water and tissue paper, and silence should be respected during the session as well. This stage is fully run by the nurse, but in rare cases, such as the family’s excessive insistence on secrecy, the nurse can ask for the help of the physician or the psychologist. Furthermore, it is extremely important that the nurse and others who conduct the session observe the following points:

- Respect the patient’s family members as well as the fact that their views toward truth-telling and patient’s autonomy could be totally different from their own.

- Verify the family’s personal, cultural and religious reasons for concealing the diagnosis from the patient as well as their concerns about truth-telling to the patient through the following questions: “Why don’t you want the patient to be informed of his/her disease?” “What do you think that I’m going to tell the patient that makes you worried?” “I know you're worried about what we are going to discuss with the patient, but we will certainly disclose any information to patients unless they themselves are not willing to receive it.” 

- Assure the patient's family that information on the diagnosis would be disclosed to the patient only to the extent that the patient requests and not beyond it. 

- Make the patient's family aware that despite their attempts to keep the diagnosis from the patient, the latter would often find out; therefore, it is the patient’s right to be informed in a suitable and appropriate way and not in an unexpected and indirect manner. 

- Explain to the family that the purpose of talking to patients is not a hasty disclosure of the diagnosis, but rather a review of their current information, recognizing their correct and incorrect understandings of the disease and knowing their level of readiness to get further informed about it. 

- Regarding the patient's religious beliefs and background, the conductor(s) of the session can benefit from Islamic teachings and their clear viewpoints on patients’ right to know about their disease and decide freely about the issues in their lives. Islamic concepts such as seeking forgiveness and making a will before death all indicate the fact that patients should be aware of their condition so that they themselves can plan for their life. 

*b. Environment preparation*

Providing the suitable environment for truth telling is also among the duties of the nurse. At this stage it is necessary to pay attention to the following points:

- A private, comfortable, clean room should be used for the purpose of disclosing the cancer diagnosis to the patient. 

- There should be no disturbing factors such as telephone or cellphone ring tones. 

- Before the session, enough chairs must be placed in the room so that all attendees including the patient, their family members and the healthcare team can take a seat. If the patient is lying on the bed, the nurse should put a chair next to his/her bed and invite the close relatives to sit as well before starting the session. 

- There must be some tissue paper, bottles of water and glasses in the room.

*c. Patient preparation*

After the preliminaries mentioned above in the previous steps, the truth-telling session to the patient and his/her family members will be held in the presence of the truth-telling team members and under the supervision of the team physician. The nurse must make the necessary arrangements to fix the time and place of the session. Before the truth-telling session, team members should have a short meeting without the presence of the patient and his/her family and review the information and the results obtained in the previous steps. When the session begins and after introducing the team members as well as the purpose of the session, the physician should ask relevant questions in order to collect a clear view of the patient’s understanding of his/her medical condition. This stage is one of the main steps of the interview and demands high concentration and good listening skills. Some of the questions that the physician can ask at this stage include:

- What information did your previous physician give you about your illness/surgery? - What do you know about your disease?

The patient’s answers to these questions should be carefully considered, because they can provide important information on his/her perception of the illness, emotional condition and the phrases and words used by him/her when speaking to the physician (for example, some patients at this stage prefer to use terms such as mass, tumor, benign or malignant, infection, anemia and so on to describe their disease).

- Physicians should not disclose the news to patients hurriedly and without preparing them. 

- If the patient pretends to be unaware of the disease or talks about it with excessive optimism that indicates denial of unpleasant realities, the physician should avoid disclosing the bad news of cancer diagnosis that day and postpone it until future sessions.

*4)    Disclosure*

Similar to the patient preparation step, the main task of disclosing the news of cancer diagnosis is up to the patient's physician at this stage. Some of the important recommendations in this regard are:

- Simple, clear and non-medical language should be used to tell the truth to the patients.

- Relevant information must be disclosed step by step and in small chunks. The physician must make sure that the patient has clearly understood the information by asking questions such as "You see what I mean?" 

- It may help to use eye contact with patients and their family members, sit close to patients and use touch techniques such as putting your hands on their shoulder or holding their hands (if the patient and the physician are of the same sex and there are no cultural barriers). 

- The physician should replace the word "cancer" with words such as "malignant mass" or "malignant tumor" when disclosing the cancer diagnosis. 

- Although disclosure of the news should be straight and clear, it is advisable to use an expression of compassion, empathy and respect when breaking the news. 

- Information about the prognosis can only be given when directly requested by the patient and his/her family, and upon establishing that the patient is ready and has the right understanding to receive it.

- The physician must avoid talking about death. If the patient or the family members need to know about the estimated time of death in order to make some important decisions, rather than giving them a definite time, for example saying, "You would survive for 6 months", the physician can give them a time range that is the average of the patients' life expectancy, such as "from some days to several weeks" or "from some months to several years."

- Information on the prognosis must be provided with an emphasis on the positive aspects rather than negative ones. In other words, the physician should highlight what can be done rather than the things that cannot be controlled by the healthcare team members.

- It is important to talk to the patient and his/her family about the uncertainty of the prognosis. For example, the physician can say, “I can just tell you things that usually happen to patients who suffer from a disease like yours, but I cannot predict what will happen to you with certainty”.

*5)    Support*

All members of the truth-telling team, especially the announcer of the bad news who is the patient's physician, play an active role in this step of the protocol. After disclosing the cancer diagnosis, the physician should try to provide sufficient emotional support to the patient and his/her family members. In some cases, after disclosing the news and answering the patient’s questions, the physician assigns the session to the nurse and the psychologist. They will in turn prepare the patient and his/her family members to properly express their emotions by providing further explanation, resolving the misconceptions, finding the source of anxiety and helping them to express their feelings more and more. 

One of the most important measures at this stage is to confirm the patient's emotions. A good technique for doing so is "empathic response" presented by Buckman (9), and consists of three main steps:

*a. Listen carefully and recognize the emotions*

The following questions can be used to discover and make sure what emotions the patient is experiencing:

- How did you feel after receiving the news?

- Did you get nervous?

*b. Identify the cause or source of the emotions*

To explore the reasons for the emotions observed in the patient, questions such as the following can be asked:

- Which part of the news you received made you more concerned?

*c. When talking to the patient, show him/her that you have been able to make a connection between the two steps above.*

Reassure the patient that his/her emotions and the reasons for them are well understood by the healthcare team, for instance by stating:

- The result of the scan was a great shock to us.

- This part of the news was certainly very disappointing.

Immediately after completing the three steps of empathic response, confirm the patient’s emotions through sentences such as the following:

- I understand that it is very difficult to accept such news 

Some other points that can be taken into account for supporting patients and families after disclosing the news are as follows:

- Beside the serious pursuit of medical treatment, the role of “prayer” and “trust in God” should be emphasized. In Iranian culture, physicians usually say that they only play an intermediary role in the preservation of human life and the will of God is above everything. 

- The patient and his/her family need to be given realistic (and not false) hope.

- After disclosing the news and if the patient and the family are willing, a counseling session may be held in the presence of the psychologist and the nurse. The purpose is to offer more emotional and spiritual support as well as complementary explanation and training to patients and their families. During this session, the psychologist can assess the concerns of the patient and the family and provide appropriate solutions, whereas the nurse can help to resolve any possible misunderstandings and give additional answers to their possible questions about the disease, treatment and the side-effects while offering emotional support. Holding such a session entirely depends on patients and their family conditions after receiving the news of cancer diagnosis. After the news is delivered by the physician, the patient or their family may be in a poor psychological state to receive further information, or conversely, they may be willing to talk about their feelings or to learn more. Patients and their families should be assured that they can have the session whenever they wish, and they should be advised on when, where, and how to contact the healthcare team members to receive the required information.

*6)    Conclusion*

At the end of the truth-telling session, the conductors can do the following to conclude the session:

- Summarize all the main points of the session and put emphasis on the most important items that were raised, especially the treatment and care programs.

- Reaffirm that the healthcare team will be present in the various stages of the disease and treatment and would not leave the patient alone.

- Urge the patient and his/her family to ask any questions they have about what has been discussed.

- Provide a summary of the essential information to the patient and his/her family members in writing.

- Before letting the patient leave the truth-telling session, especially when the session is held at the physician’s office and the patient is not hospitalized, verify his/her safety; for instance, see if they can drive home safely, or if there is any risk of committing suicide.

## Discussion

Perception of bad news is influenced by the beliefs and attitudes of each society. Thus, developing localized protocols tailored to each community's cultural infrastructure, and training healthcare teams on how to use these guidelines can be a valuable step toward a more effective implementation of the truth-telling process to the patients.

Some steps in this protocol (including patient and environment preparation, disclosure, support and conclusion) are similar to those in other breaking bad news protocols that have been developed in other countries. Examples of these protocols and guidelines are “Setting; Perception; Invitation; Knowledge; Empathy; Strategy and Summary” (SPIKES) (9), “Interview; Gather; Assess and Achieve; Decide, Disclosure and Discuss” (IGAD)(2), “Background; Rapport; Explore; Announce; Kindling and Summarize” (BREAKS) (21), and the guideline for breaking news of prognosis and end-of-life to adults in advanced stages of a life-limiting illness (22). The above-mentioned steps are similar in principles such as: investigating patients’ awareness of their disease (9, 2, 21, 22); assessing patients’ willingness to get informed about their illness (9, 2, 22); providing the appropriate setting for diagnosis disclosure (9, 21, 22); disclosing the bad news using a simple, non-technical language and a step-by-step process with respect and empathy (9,2, 21,22); offering emotional support after the news disclosure (9,2,22); and summarizing the provided information (9, 21,22). It should be noted that despite these similarities, there are significant differences between the current protocol and Western guidelines, some of which will appear below.

The researchers believe that one of the most important differences between this protocol and the protocols in Western countries lies in the consideration for the views of patients’ close relatives in informing the former about their illness. Given the priority of the principle of “respect for autonomy” in Western communities, consideration for the relatives’ views is not common in the protocols and guidelines developed in those countries. In Asia and the Middle East, however, the principle of “no harm” is superior to the principle of “respect for autonomy” and the family plays a very important role in deciding whether or not to inform the patient (14); therefore, it is essential in Iran to consider the opinions of patients’ close family members as well when breaking the news, and this has been included in the present protocol. In their guideline for breaking bad news in Muslim societies, Salem and Salem refer to the family and their efforts to conceal the diagnosis from the patient in the assessment phase, but ultimately leave it to the physicians to decide whether, to what extent and to whom to disclose the diagnosis (2). In the present protocol, in addition to the “assessment” phase, the two steps of “planning” and “family preparation” are included. Moreover, the researchers have made an effort to respect the patients’ wishes regarding whether or not to disclose the diagnosis to them. If the patient requests to be informed, but the family members are opposed to truth-telling, then attempts are made to obtain their consent for disclosure of the diagnosis to the patient by holding a family meeting. 

Replacing the term “cancer” with less negative words like “malignant mass” or “malignant tumor” when disclosing the bad news is one of the items in the current protocol that contradicts the western guidelines’ emphasis on avoiding euphemism. This is because of significant cultural differences between Western and non-Western countries. In Eastern and Asian countries, the word “cancer” is associated with misconceptions such as incurable and fatal diseases, and is a harbinger of fear, anxiety and suffering for individuals (23-26). Accordingly, the findings of various studies in these countries, including Iran, show that there is a tendency to avoid using the word “cancer” among patients, their families and members of the healthcare team. Moreover, they prefer to receive the cancer diagnosis news indirectly and with less-negatively charged words, for instance mass or tumor. It is generally believed that euphemism reduces the distress caused by the news and induces a positive feeling in the patient because of the emotional support offered by the physicians, and helps them to have an easier transition from health into the disease (21-23, 26-28). Narayanan et al. also approves euphemism for telling the truth to the patient, stating, “Using euphemism is a good technique, but it should not cause any confusion and uncertainty in the patient” (21).

Not mentioning death and its approximate time when disclosing bad news is another principle emphasized in the present protocol. In Iran, due to cultural and religious reasons, most people prefer that the physician not talk about death while disclosing their diagnosis. The reason for people's unwillingness to talk about death may be attributed to their beliefs and attitudes. Muslims believe that despite all the efforts made by the healthcare team to treat and control the disease, death and life are almost completely in the hands of the Lord and it is only He who has the divine power to determine the time of death. Therefore, they prefer not to discuss this otherworldly phenomenon which humans have not much control over. Attar and Malekian state, “Some people’s religious beliefs cause them to consider it inappropriate for physicians to comment on the life span of the patient or how long they expect the patient to survive, and would get annoyed if physicians discusses such issues” (28). 

Emphasizing religious principles while disclosing the bad news by members of the healthcare team was among other issues underpinned in the present protocol. Different sources confirm that the tendency toward religion and spirituality is an important adaptive strategy that contributes to patients’ better compatibility with their illness. In addition, the spiritual health of cancer patients is directly related to their lower depression, greater enjoyment of life, higher quality of life, and less disappointment at the late stages of their disease (28-30). There is no denying the positive impact of spiritual and religious beliefs on the psychological state of individuals in difficult moments of life including the crisis following a cancer diagnosis. Therefore, it is recommended that physicians and nurses try to benefit from these beliefs, even in terms of positive and optimistic sentences such as "Everything is in the hands of God" and "God is merciful" when telling the truth (2, 16 (. Although the protocols already developed by Western countries put emphasis on investigating the individual, cultural, or religious backgrounds of the patient before breaking the bad news, they do not recommend using religious phrases to disclose the news (13).

## Conclusion

The protocol presented in this article is a local guideline for training healthcare team members as well as implementing the process of bad news disclosure to cancer patients in Iran. This protocol consists of six main steps: assessment, planning, preparation, disclosure, support and finally conclusion. In developing the present protocol, the researchers did their best to take into account the religious and cultural viewpoints of the Iranian society toward cancer and its consequences, as well as the differences between Iran and Western countries. In addition, another strong point of this protocol is that its implementation is based on inter-professional team collaboration.

It is hoped that this protocol could be manipulated as a useful guideline in training the members of the healthcare team, and through its effective implementation in disclosure of bad news to cancer patients, reduce the many complications caused by hearing such adverse news.
